# Strip Adjustment of Airborne LiDAR Data in Urban Scenes Using Planar Features by the Minimum Hausdorff Distance

**DOI:** 10.3390/s19235131

**Published:** 2019-11-23

**Authors:** Ke Liu, Hongchao Ma, Liang Zhang, Zhan Cai, Haichi Ma

**Affiliations:** 1 School of Remote Sensing and Information Engineering, Wuhan University, Wuhan 430079, China; Liu_ke@whu.edu.cn (K.L.); mahaichi@whu.edu.cn (H.M.); 2 Department of Oceanography, Dalhousie University, Halifax, NS B3H 4R2, Canada; 3 Faculty of Resources and Environmental Science, Hubei University, Wuhan 430062, China; zhangliang@hubu.edu.cn; 4 School of Resources Environment Science and Technology, Hubei University of Science and Technology, Xianning 437000, China; zhan.cai@whu.edu.cn

**Keywords:** strip adjustment, building and roof plane segmentation, building and roof plane matching, Minimum Hausdorff Distance (MHD)

## Abstract

In Airborne Light Detection and Ranging (LiDAR) data acquisition practice, discrepancies exist between adjacent strips even though careful system calibrations have been performed. A strip adjustment method using planar features acquired by the Minimum Hausdorff Distance (MHD) is proposed to eliminate these discrepancies. First, semi-suppressed fuzzy C-means and restricted region growing algorithms are used to extract buildings. Second, a binary image is generated from the minimum bounding rectangle that covers overlapping regions. Then, connected components labeling algorithm is applied to process the binary image to extract individual buildings. After that, building matching is performed based on MHD. Third, a coarse-to-fine approach is used to segment building roof planes. Then, plane matching is conducted under the constraints of MHD and normal vectors similarity. The last step is the calculation of the parameters based on Euclidean distance minimization between matched planes. Two different types of datasets, one of which was acquired by a dual-channel LiDAR system Trimble AX80, were selected to verify the proposed method. Experimental results show that the corresponding planar features that meet adjustment requirements can be successfully detected without any manual operations or auxiliary data or transformation of raw data, while the discrepancies between strips can be effectively eliminated. Although adjustment results of the proposed method slightly outperform the comparison alternative, the proposed method also has the advantage of processing the adjustment in a more automatic manner than the comparison method.

## 1. Introduction

Airborne LiDAR technology has been becoming an indispensable tool regarding three-dimensional (3D) geospatial data acquisition for urban applications [1], such as road detection [2], building extraction [3] and 3D reconstruction [4], population estimation [5], 3D change detection [6], assessment of post-disaster building damage [7], and many others [8].

**Figure 1:**
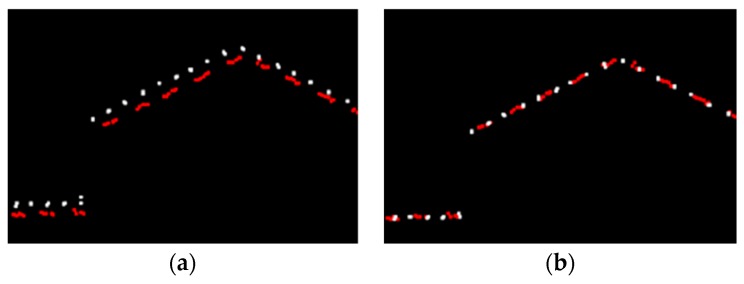
Discrepancies before and after strip adjustment observed from profiles: (**a**) Before strip adjustment; (**b**) After strip adjustment.

In practice, LiDAR data are collected by parallel flight strips where the region of a single strip is generally much less than the entire region being surveyed. Thus, multiple strips are required and should be stitched together so that the whole region can be covered. This operating fashion is very similar to the conventional aero-survey by photogrammetric technique, and partial overlapping between adjacent strips is required to mosaic data from multiple strips into an integrated dataset. The lateral overlap can vary from 10% to 30%, depending on the geomorphological characteristics of the region being surveyed [9,10]. A LiDAR system needs to be calibrated by both the system provider before the equipment is shipped, and again by the end-user before data collection, in order to remove most of the systematic errors caused by individual components and their integration [11,12]. No discrepancies between adjacent strips should occur if there are neither systematic errors nor random errors existing. However, this is impractical due to missing or improper calibration and operational procedures. In some cases, small discrepancies may persist even though the system has been carefully calibrated. Figure 1 shows the discrepancies between adjacent strips, though system calibration has been performed. Therefore, strip adjustment, a process that can eliminate discrepancies between adjacent strips, is necessary if high accuracy final products, such as Digital Elevation Model (DEM), are required from the airborne LiDAR point cloud.

Many research works have been conducted in the past two decades regarding strip adjustment. Some methods combine other data sources such as optical images [13,14], but methods based solely on point cloud were the mainstream focus [15,16,17,18,19,20,21,22,23,24,25]. Such methods could broadly be categorized into two classes: with and without ground control points. In the adjustment with ground control points, corresponding points distributed in adjacent strips must first be extracted, then parameters for a given adjustment model are calculated based on the corresponding points; this is similar to the photogrammetric block adjustment of independent models [15,16]. However, to extract corresponding points is difficult because laser scanning points are discrete and irregular in space. Many point detection algorithms developed for optical images cannot be employed without modification. Thus, artificial landmarks are setup in some applications. Though these are feasible for system calibration, which is usually performed in a pre-setup test site, artificial landmarks can hardly be setup over the whole area of a real project.

Strip adjustment without control points, on the other hand, adopts corresponding geometrical features, including points, line segments, planes, etc., to calculate the transformation parameters between adjacent strips. Considering that there are large numbers of buildings in urban scenes and the roofs of these buildings can be viewed as planes, algorithms based on corresponding planes were developed instead of corresponding points, thus overcoming the difficulties of detecting point features in an irregular point cloud. Wu and Fan [17] used the OpenStreetMap-aided method to select simple-structured roof planes as the corresponding features and then normal vectors of them were calculated and input into an over-determined mathematical model. The transformation parameters were then estimated through the given model. Habib et al. [18] used LiDAR intensity images of overlapping strips for patch selection, from which linear features were generated. Although tie planes can be detected by using above-mentioned methods, it is time-consuming and sometimes impractical because some auxiliary tools or data, such as OpenStreetMap, were required in the process. Moreover, merely specific structures can be detected accurately, such as gables or flat roofs, resulting in missing or failure detection of many other planar patches. In Sande et al. [19], points in overlapping strip areas were interpolated into raster format by using Inverse Distance Interpolation, then corresponding gable planes were detected based on the gridded data. But it requires extra computation to transform point data into raster format because the selection criteria for corresponding planes could not be applied to the discrete points directly. In Pfeifer et al. [20], both ground and building points were segmented in one strip, then points in the adjacent strip corresponding to the segments were automatically selected under two criteria: points from one strip have to be surrounded by points from segments in the adjacent and the vertical distances between points and corresponding surface should be within limited range. In other words, in the method, the number of points of adjustment planes in one strip must be less than or equal to the other, which limits its capability in detecting tie surfaces. Their experiments showed that the algorithm efficiency would be improved if tie surfaces were extracted merely from building points. Filin [21] studied the influence of surface characteristics on strip adjustment and experimental results revealed that using sloped surfaces with different orientations helped improve the adjustment result.

Lines are the other geometric features that have been widely adopted for strip adjustment. Lee et al. [22] and Habib et al. [23] used the linear features generated from building roofs with ridge lines in overlapping region for adjustment computation. Since planar, linear and point features were all suitable for adjustment, several methods combining these features for the estimation of transformation parameters were proposed. In Rentsch and Krzystek [24], roof ridge lines generated from roof plane intersection and roof planes were both used in the adjustment. In Kilian et al. [15], roof planes and corner coordinates of buildings were considered. In Zhang et al. [13], planar, linear and point features were all adopted in the adjustment computation. Their experimental results demonstrated the advantages of their models, but to extract all these features, especially the point features, poses a challenging task.

TerraMatch is a commercial software that is widely adopted for system calibration and strip adjustment. It computes parameters for calibration and adjustment from overlapping flights with trajectory files as one of the inputs [25], which limits its applicability because trajectory files are not always available to end-users, for instance, when data collection and data application are performed in separate companies.

Note that the discrepancies between strips are normally not that much after system calibration and, considering that there are lots of buildings in urban scenes, a strip adjustment using planar features detected merely from building points is proposed in this paper. It composes four principle steps: First, progressive Triangle Irregular Network (TIN) densification (PTD) is performed on the raw point cloud so that the raw dataset is classified into ground and non-ground subsets [26,27]. Semi-suppressed fuzzy C-means and restricted region growing algorithms are then applied to extract laser footprints reflected from buildings from the non-ground subset [28]. Second, building footprints are converted to a binary image at first, then connected components labeling algorithm is applied to segment the binary image [29], resulting in footprint clusters corresponding to individual buildings. Building matching is then performed by MHD to extract corresponding pairs of buildings. Third, a coarse-to-fine approach is proposed to segment roof planes from the matched buildings, and plane matching is performed by the combination of MHD and normal vectors similarity, resulting in matched roof planes. The fourth step is the calculation of the parameters of the adjustment model based on the matched roof planes, constrained by the Euclidean distance minimization between pairs of corresponding planes located in adjacent strips. Point cloud is transformed in a strip-by-strip manner, resulting in a mosaic dataset of the whole surveying area.

The main contributions of this work can be summarized as follows: (1) Minimum Hausdorff Distance (MHD) is introduced to detect corresponding planes from building points in an automatic manner without limiting building structures or transforming point data into gridded data or using auxiliary data. (2) A coarse-to-fine segmentation of building points is proposed specifically for the purpose of planar features extraction, which can detect enough planar patches for adjustment computation. (3) Airborne LiDAR data acquired by a dual-channel LiDAR system are used to verify the proposed method, as well as the single channel LiDAR data. Compared with the methods that select corresponding planes in a manual manner, our method can automatically detect enough and correct corresponding planes for adjustment.

The rest of the paper is organized as follows: Section 2 introduces the algorithm flow, mainly including building segmentation and matching, roof plane segmentation and matching, and adjustment model. The procedures of building and roof planes segmentation and matching are described in specific detail. Section 3 presents and analyzes the experimental results. Section 4 summarizes the proposed method and discusses the limitations of the proposed method.

## 2. Methodology

**Figure 2:**
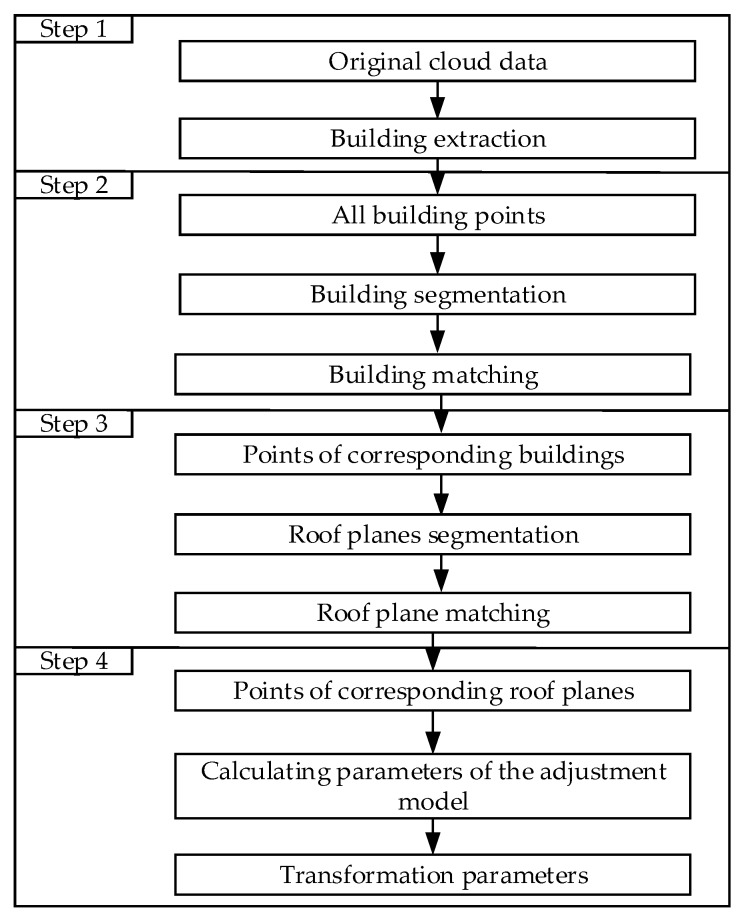
The workflow of the proposed method.

The proposed method mainly includes four steps: building extraction from non-ground points, building segmentation and matching, roof plane segmentation and matching, and transformation parameters calculation. The algorithm workflow is shown in Figure 2.

### 2.1. Building Segmentation

Building segmentation is used to categorize the laser footprints reflected from buildings into multiple clusters, each of which represents an individual building. To achieve this purpose, raw point cloud is firstly filtered by progressive TIN densification (PTD). PTD is a widely employed filtering method by both the academic community and engineering applications because of its accuracy and efficiency, and it has been successfully integrated with commercial software, such as Terrasolid and LiDAR_Suite [26,27]. Then buildings are extracted from the non-ground points by semi-suppressed fuzzy C-means and restricted region growing algorithms proposed by the authors [28]. It consists of two main steps: (a) seed points identification of building roofs by the semi-suppressed fuzzy C-means; (b) restricted region growing to search for more building points.

The dataset of building footprints is at this stage a single undifferentiated set of points. It should be segmented into different clusters corresponding to individual buildings, so that building matching can be conducted on the individual building segments. Considering the complex roof structures of buildings and the high computational efficiency required in practice, the dataset is converted to a binary image at first, then a digital image processing method is introduced to segment individual buildings. Converting the three-dimensional (3D) point cloud into a two-dimensional (2D) image and then using matured image processing algorithms for feature extraction, classification etc., is a commonly employed strategy in point cloud processing [29,30,31,32]. In the paper, the dataset of building footprints is converted into an image by the following steps: First, the minimal and maximal values of *x* and *y* coordinates of the building footprint dataset in the overlapping region are determined and denoted by $x_{min}$, $x_{max}$, $y_{min}$ and $y_{max}$. Then the minimum bounding rectangle that covers the overlapping region is partitioned into uniform cells with size *l*, where *l* is a parameter related to point cloud density of the dataset. Therefore, a total number of $[x_{max}-x_{min}]/l$ and $[y_{max}-y_{min}]/l$ cells are generated. Set the value binary 1 if there are building points within a cell and binary 0 otherwise. A binary image can be obtained in this way. Connected components analysis of the generated binary image is performed to segment the individual buildings, which is one of the classical digital image processing methods specifically developed for binary image segmentation. Despite its long history, it is still one of the research hotspots in image processing and many improvements have been achieved [33]. It consists of the connected components labeling and decision making. The connected components labeling changes connected pixels to regions. All pixels that have the value binary 1 and are connected to each other by a path of pixels all with value binary 1 are given the same identifying label. A path is defined by 4- or 8-neighbors of pixel. Different definition results in slightly different regions. The label is a unique name or index of the region to which the pixels belong [34]. The algorithm proposed by Di and Bulgarelli [35] is adopted in the paper for its efficiency. It consists of two subsequent scans of the input image. After the first scan, no temporary label is assigned to pixels belonging to different components, but different labels may be associated with the same component, which were registered as equivalent classes and were further processed in the second scan. Many improvement algorithms were developed regarding the second scan in order to improve the efficiency of the algorithm. In [35], equivalences are processed directly in the first scan so that equivalence classes are always maintained to be updated during the scan. This is obtained by associating a new equivalence class with each new label and by merging the corresponding classes as soon as a new equivalence is found.

### 2.2. Building Matching

Building segmentation is followed by building matching in our flowchart, in which points of individual corresponding buildings in the adjacent strips are to be matched. Given a model represented by 3D point cloud and searching for the matched one from a model library is quite challenging, and the similarity that indicates the degree of resemblance of 3D point sets is expected to be a hot topic. Hausdorff Distance (HD) is a frequently used similarity measurement. It can quantify the similarity between two arbitrary point sets without the necessity of establishing the correspondence between points and has been proved to be an efficient measure for image and point cloud matching [36,37,38]. Thus, it was employed for the matching process in the paper.

HD is a MAXMIN distance measure between two point sets. Given two finite point sets $A=\left\{a_{1},..,a_{m}\right\}$ and $B=\left\{b_{1},..,b_{n}\right\}$, the HD is defined as

(1) H(A,B)=max(h(A,B),h(B,A))

where $h\left(A,B\right)=\underset{a\in A}{max}\underset{b\in B}{min}\left|\left|a-b\right|\right|$ and $h\left(B,A\right)=\underset{b\in B}{max}\underset{a\in A}{min}\left|\left|a-b\right|\right|$. The ||·|| is any norm distance metric, and Euclidean norm was adopted in the paper. The function h(A,B) is called the directed HD from A to B. Intuitively, if h(A,B)=d, then each point of A must be within a distance d of some point of B [39], or equivalently, it means that every point in A is at most h(A,B) away from B.

In practice, taking the maximum of all the distances is dangerous because possible noise (outliers) in one set can then greatly impact the Hausdorff Distance. Fractional Hausdorff Distance, in which some percentage (say 90%) of the points in *A* have the distance or less to some point in *B*, is an alternative to overcome outliers. Because outlier removing is one of the preprocessing steps in airborne LiDAR processing, conventional Hausdorff Distance can be employed without danger in the present research. Computational efficiency is another issue that must be considered in practice. For fixed sets *A* and *B*, Hausdorff Distance can be computed in time using O((n+m)log(n+m)) [40]. More efficient computational strategies have been explored in recent years [41,42] when the number of the points in A or B is very large, say more than 10 million in each. But in the present application, the number of laser points from an individual building is limited. For instance, suppose a dataset has average point density of 10 points/m^2^, then a building with an area of 500 m^2^ has 5000 points. Thus, computational efficiency is a less challenging problem in the current scenario.

Armed with the Hausdorff Distance, building matching was performed as follows: a minimum bounding rectangle was determined by overlay analysis in the overlapping area of adjacent strip. Buildings within the rectangle were retained for the matching. For an individual building P in one strip, calculate HD between P and all individual buildings in the adjacent strip. The building pair with MHD was identified as matched one.

### 2.3. Roof Plane Segmentation

The mathematical model in our strip adjustment was based on the minimization of the distance between corresponding planar patches. Therefore, roof planes need to be segmented at first, then planar patch matching is performed. A coarse-to-fine strategy was proposed for the segmentation, which consisted of preprocessing and segmentation and were described below. Notice that all the following steps were performed only on the point datasets of corresponding buildings.

#### 2.3.1. Preprocessing the Point Cloud of Buildings

Preprocessing step is specific for subsequent roof plane segmentation, which contains the calculation of normal vector, curvature, and planar equation for a given building point based on its *k* neighborhood. Eigenvectors and eigenvalues were calculated based on *k* neighborhood, which were denoted by $\vec{v_{1}}$, $\vec{v_{2}}$,$\;\vec{v_{3}}$, and the eigenvector corresponding to the smallest eigenvalue $\lambda_{1}$ is the normal of the plane associated with the given point. Hence a point-normal form equation of the associated plane is defined. The curvature at a given point can be calculated based on above eigenvalues [43]. Normal vectors of all building points were normalized to unit vectors. To speed up the process of searching *k* nearest points of a given building point, KD-tree was constructed for the whole dataset before the preprocessing step.

#### 2.3.2. Coarse-to-Fine Segmentation Algorithm

The idea behind the plane segmentation is based on the fact that buildings are generally composed of several planar patches. Points on the same roof planes in adjacent strips satisfy the same planar equation and hence have similar normal vectors. Therefore, under the constraints of normal vector similarity and point-to-plane distance, roof planes were segmented from building points.

This step consists of the following sub-steps:

Step 1: Select the point corresponding to the smallest curvature as the seed point of a roof planar patch from the dataset *Q* of a building, denoted by $P_{seed}$. Construct a stack and add $P_{seed}$ to the end of the stack. For each $P_{other}\in Q,P_{other}\neq P_{seed}$, calculate the cosine similarity between *P_seed_* and *P_other_*, and the distance from *P_other_* to the plane associated with the seed point, denoted by *Cs* and *Ds* respectively. If $C_{S}\geq C_{s0}\;\mathrm{and}\;D_{s}\leq D_{s0}$, where $C_{s0}$ and $D_{s0}$ are two predefined thresholds, then add the point *P_other_* to the stack; otherwise, *P_other_* is labeled as *remaining*. All *remaining* points construct dataset *Q_remaining_*.

Step 2: If *Q_remaining_* is non-empty, then repeat sub-step 1 for *Q_remaining_* so that another *Q_remaining_* is formed. The process is continued until all the points in the original set *Q* are clustered to different *Q_remaining_* sets.

Step 3: Choose the *Q_remaining_* with largest number of elements and fit a plane equation by least square method. For each point *P* in Q, calculate the distance from *P* to the fitted plane. If the distance is less than a predefined threshold $D_{p0}$ then *P* is considered to lie on the plane. Otherwise, it is categorized into the unsegmented point set *Q_unsegmented_*.

Step 4: If *Q_unsegmented_* is non-empty, then go to sub-step 1 with *Q* = *Q_unsegmented_*. Otherwise, stop.

Four thresholds were predefined in the above algorithm, including the preprocessing step: *k* for the size of nearest neighborhood, $C_{s0}$ for cosine vector similarity and $D_{s0}$, $D_{p0}$ respectively for distance thresholds. Though these parameters influence the final segmentation results, they are far more sensitive to small roof patches than to large ones. Therefore, large roof plane is used in the strip adjustment. Because the planar patches are used for the establishment of adjustment model, rather than for 3D building reconstruction, we argue that the above procedure is effective for our present purpose.

### 2.4. Roof Plane Matching

Bearing in mind that planar patches are merely used for adjustment model establishment, in practice, only patches with enough footprints remained for the calculation of the parameters of the adjustment model, because plane fitting with an insufficient number of points is unreliable. Moreover, computational efficiency shall be improved by ignoring some planar patches with a small number of footprints. The optimal number of footprints for adjustment computation is point-density oriented and will be discussed in the next section. Therefore, only large planar patches were retained for roof plane matching.

The matching of two roof planes in adjacent strips was performed based on two similarity measures: Minimum Hausdorff Distance (MHD) and cosine similarity between the normal vectors of the two planes in order to improve the robustness of matching where complex structures occur in matched buildings. The specific matching process can be described as follows: (a) For a roof plane p of a given building Q, calculate HD between p and all roof planes of the corresponding building in the adjacent strip, denoted by *Q_adjacent_*. (b) The roof plane with the minimum HD, denoted by p_adjacent_, is taken as the matched plane of p if its cosine similarity is larger than the predefined threshold. (c) Repeat the above steps for the rest planes of Q until all corresponding planes are detected. (d) Repeat (a), (b) and (c) for all other corresponding buildings to detect their respective corresponding planes.

### 2.5. Mathematical Model for Adjustment Computation

Several adjustment models exist in literature which can be categorized as: one-dimensional adjustment model [16,44], three-dimensional adjustment model [45], rigid body transformation adjustment model [23,46], and rigorous system-driven adjustment model that considers the systematic errors [47]. However, the application of a system-driven model requires an input of system observations and these are not usually available to the end user [48]. An optimum solution for adjustment should be practical and assume the existence of data normally available to a user as well as compensating for actual errors in the system [49]. By assuming that the system calibration has been performed to a LiDAR before data acquisition, the rigid transformation model was adopted to describe the misalignment in the overlapping region between adjacent strips, which has been widely used in photogrammetry and extensive literature has demonstrated its validity [17,18,23,46,48].

Two point sets from adjacent strips corresponding to a same roof plane can been detected after the roof plane matching. If there were no misalignments in three dimensions between the adjacent strips, then the two planes fitted from the point sets should be identical. Because a rigid transformation model is characterized by a rotational matrix and a translation vector, the parameters should satisfy the constraints that the Euclidean distance between the two fitted planes should be minimal. Denoting the rotational matrix and translation vector between adjacent strips by R(φ,ω,κ) and T respectively, where φ, ω, κ are three rotational angles which satisfies det (R(φ,ω,κ)) = 1 [17]. Then, a point set $P=\{{\left(x_{i},y_{i},z_{i}\right)}^{T},\;i=1,2,\cdots \;\mathrm{the}\;\mathrm{total}\;\mathrm{number}\;\mathrm{of}\;\mathrm{points}\;\mathrm{in}\;\mathrm{the}\;\mathrm{set}\}$ can be expressed by R(φ,ω,κ)P+T after being transformed. For a given transformed point in one of the roof point set, it should lie on the plane fitted by the other roof point set in the adjacent strip, therefore, Equation (2) can be written as:

(2) JH=0

where $J=\left(n_{x},n_{y},n_{z},-d\right)$ are coefficients of the plane equation with normal $n=\left(n_{x},n_{y},n_{z}\right)$ and distance d to the origin, $H=\left[\begin{array}{c} R\left(\varphi ,\omega ,\kappa \right)P+T\\ 1 \end{array}\right]$. For the convenience of subsequent calculations and coding R(φ,ω,κ) is rewritten by three row vectors $r_{1}^{T}$, $r_{2}^{T}$ and $r_{3}^{T}$: $R\left(\varphi ,\omega ,\kappa \right)=\left[\begin{array}{c} r_{1}^{T}\\ r_{2}^{T}\\ r_{3}^{T} \end{array}\right]$. Let $T={\left[\begin{array}{ccc} t_{x} & t_{y} & t_{z} \end{array}\right]}^{T}$, where $t_{x}$,$\;t_{y}$ and $t_{z}$ denote the translation parameters along x, y and z axis. Then Equation (2) can be rewritten as:

(3) $$n_{x}\left(r_{1}^{T}P+t_{x}\right)+n_{y}\left(r_{2}^{T}P+t_{y}\right)+n_{z}\left(r_{3}^{T}P+t_{z}\right)=d$$

A system of linear equations can be formed for *m* points and corresponding planes:

(4) $$\left[\begin{array}{cccccc} n_{x}^{1}P^{1} & n_{y}^{1}P^{1} & n_{z}^{1}P^{1} & n_{x}^{1} & n_{y}^{1} & n_{z}^{1}\\ \ldots & \ldots . & \ldots & \ldots & \ldots & \ldots \\ n_{x}^{m}P^{m} & n_{y}^{m}P^{m} & n_{z}^{m}P^{m} & n_{x}^{m} & n_{y}^{m} & n_{z}^{m} \end{array}][\begin{array}{c} r_{1}\\ r_{2}\\ r_{3}\\ t_{x}\\ t_{y}\\ t_{z} \end{array}]=[\begin{array}{c} d^{1}\\ \ldots \\ d^{m} \end{array}\right]$$

For all the corresponding planar patches, forming the Equation (4) and least mean squares estimator was applied to estimate the rotation matrix and the translation vectors. The above adjustment process is performed for each pair of adjacent strips respectively when dealing with multiple strips. Mean square error σ was adopted to indicate the precision of the estimated parameters, which can be calculated by using the following formula:

(5) $$\;\sigma^{2}=\frac{V^{T}V}{m-t}$$

where V is residual vector that actually contains the remaining point-to-plane distances after the adjustment. t is the number of unknown parameters in the adjustment. Note that the weight matrix used in adjustment is a unit matrix by assuming that all the points used to estimate transformation parameters have the same accuracy. A smaller σ indicates increased precision of the parameters.

## 3. Results

Two different types of datasets, one which was acquired by a conventional single-channel LiDAR and the other which was acquired by a dual-channel system, were selected to validate the proposed method, which was implemented by C++ and the results derived from it were displayed by LiDAR_Suite, an airborne LiDAR data processing software developed by the Research and Development (R&D) group of the authors. Besides, method in Wu and Fan [17] was cited for the purpose of comparison in two experiments, and commercial software TerraMatch was used for comparison in experiment of single channel LiDAR data.

### 3.1. Experiment of Single Channel LiDAR Data

#### 3.1.1. Data Description

**Figure 3:**
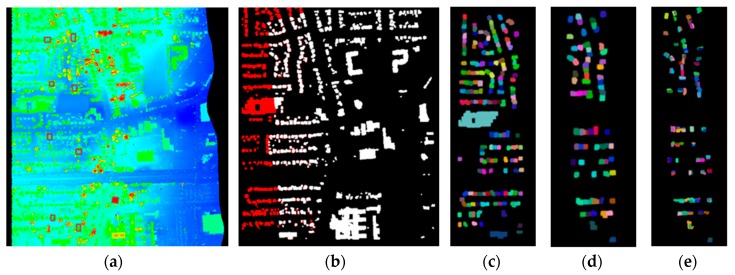
One of the adjacent strips in matching and segmentation experiment: (**a**) Part of adjacent strips of point cloud (rendered by height); (**b**) Buildings extracted by the proposed method in the paper. Red and white represent buildings in different strips; (**c**) Building segmentation of one strip of the adjacent strips. Results were rendered in different colors; (**d**) Buildings matched. Matched buildings were rendered in different colors; (**e**) Distribution of the corresponding patches for adjustment.

Seven parallel strips were acquired by airborne LiDAR in the city of Niagara, where six groups of adjacent strips can be formed. Though manual calibration has been performed, discrepancy between adjacent strips remained. The average flying height is about 1200 m, average flying speed is 60 m/s, average strip width is 700 m and average lateral overlap between adjacent strips is about 20%. The average point density of these two strips is about 3.3 points/m^2^, which means a building with a floor area of 100 m^2^ contains approximately 350 laser points. Part of two adjacent strips are illustrated in Figure 3a, which was rendered by the height values. Typical objects in the scene include buildings, roads, grasses and trees.

#### 3.1.2. Results of Building Matching and Planar Patch Segmentation

Semi-suppressed fuzzy C-means and restricted region growing algorithms were applied to extract building points from non-ground points, which were acquired by filtering the original laser dataset by PTD. Figure 3b illustrates the buildings extracted from the region displayed in Figure 3a, where red and white represent building footprints from the two adjacent strips. Then, connected components labeling algorithm was used to process the binary image generated from them to segment individual buildings. Segmentation results of one of the strips were displayed in Figure 3c, where individual buildings were rendered with a specific color. In order to save matching time, only buildings containing 100 to 400 footprints were retained for building matching. Minimum Hausdorff Distance was set to 10 meters empirically, which is the threshold to determine if two individual buildings are matched. Figure 3d illustrates the matching results, where individual matched buildings are rendered with randomly chosen colors. There were 70 pairs of buildings matched in the two strips displayed in Figure 3.

**Figure 4:**
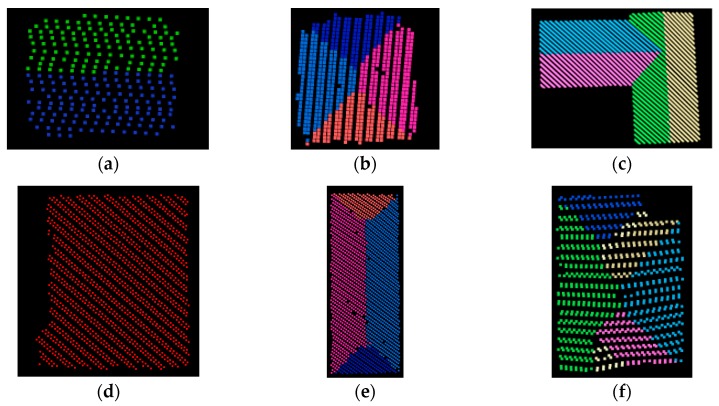
Segmentation results of planes from six types of specific roof structure by the coarse-to-fine method: (**a**) Gable; (**b**) Pyramid; (**c**) T-Joint; (**d**) Flat; (**e**) Hip; (**f**) Complex structure.

In roof plane segmentation, optimal values for parameters k, $C_{s0}\;$, $D_{s0}$, and $D_{p0}$ were determined by referring to [43] and trial and error: k = 15, $C_{s0}\;$= 0.95, $D_{s0}\;$= 0.4 m, and $D_{p0}\;$= 0.1 m. These values were confirmed to be optimal in experiment 2 where the point density is 2.5 points/m^2^, as stated in the next subsection. Six types of buildings with specific roof structures, such as gable, T-joint, flat, etc. [50], were selected to illustrate the segmentation results, as shown in Figure 4, where different colors represent different planar patches.

It is obvious that building roofs can be successfully segmented into different patches by using the proposed coarse-to-fine method, regardless of whether the roof structure is simple or complex. Roof planes with more than 60 points and cosine similarity larger than 0.96 were retained for adjustment computation, therefore, as for the adjacent strips illustrated in Figure 3, 114 pairs of corresponding planes were matched, as shown in Figure 3e.

#### 3.1.3. Parameters Estimation and Evaluation

Adjacent strips displayed in Figure 3 were chosen to illustrate the comparison of the rotation matrix and translation vector estimated from the proposed method and the one in Wu and Fan [17]. Because corresponding roof planes were manually selected in Wu and Fan [17], eight pairs of such patches were selected for the estimation of the rotation matrix R(φ,ω,κ) and translation vector T. The approximate locations of these patches were indicated by the small rectangles in Figure 3a. A comparison between the two methods with respect to estimated transformation parameters, σ, the number of corresponding planes and the manner of selecting corresponding planes was listed in Table 1.

Table 1 shows that both R(φ,ω,κ) estimated from two methods are close to each other due to the fact that they are approximate to the unit matrix. The magnitude of the difference between the two translation vectors is small too. Such results are rational because most deviations were eliminated after system calibration, leaving merely slight discrepancies existing between adjacent strips. Although there are slight differences between two methods in transformation parameters estimation, there were 114 pairs of corresponding planes for adjustment computation automatically detected by our method, which was far more than the comparison method where 8 matched planar patches were selected manually. Moreover, the mean square errors of the proposed method were slightly less than the comparison one, indicating the overall accuracy of the parameters estimated by our method is better than the comparison method.

In order to visually evaluate the accuracy of strip adjustment, eight profiles before and after adjustment were selected (small rectangles in Figure 3a), as shown in Table 2. Average distance $D_{mean}$, which was defined as the average distances from the points in one strip to the fitted plane in the adjacent, was introduced to quantitatively evaluate the adjustment results. Root Mean Square Error (RMSE) of $D_{mean}$ was calculated by assuming that the distances between matched planes were zeros after adjustment. Moreover, the reduced RMSE $\left(N_{RMSE}\right)$, denoted by $\left(RMSE_{before}-RMSE_{after}\right)/RMSE_{before}$, where $RMSE_{before}$ and $RMSE_{after}$ represented RMSE before and after adjustment respectively, were listed in the Table 2. Larger $N_{RMSE}$ indicates the increased precision of the parameter estimation. Points within the red rectangles in the first column of Table 2 were adopted to calculate $D_{mean}$. It is obvious that discrepancies between adjacent strips exist, even though system calibration was performed to the original dataset, indicating the necessity of strip adjustment. Accuracy of the adjustment results of the proposed method, measured by $D_{mean}$, RMSE and $N_{RMSE}$ are slightly better than the comparison one. The $N_{RMSE}$ is improved 0.8% and 0.8% by the proposed method compared with the comparison and TerraMatch. Considering that our method segments and matches buildings and planar patches in an automatic fashion, it outperforms the method in Wu and Fan [17]. Moreover, though some predefined thresholds are required in the roof plane segmentation and matching stages, and these threshold values affect the final adjustment more or less, the proposed method dose achieve satisfying results. The final adjustment results of the seven strips were listed in Table 3.

### 3.2. Experiment of Dual Channel LiDAR Data

#### 3.2.1. System and Data Description

**Figure 5:**
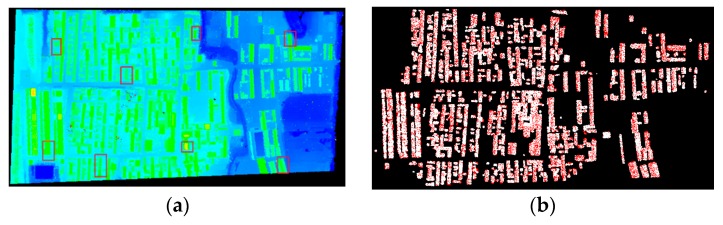

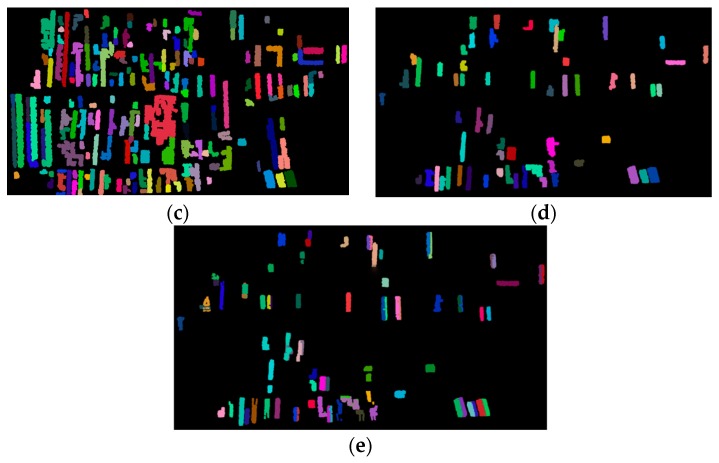
One of the adjacent strips in matching and segmentation experiment: (**a**) Part of adjacent strips of point cloud (rendered by height); (**b**) Buildings extracted by the proposed method. Red and white represent buildings in different strips; (**c**) Building segmentation of one strip. Rendered in different colors; (**d**) Buildings matched. Individual buildings were rendered in randomly chosen colors; (**e**) Distribution of the corresponding patches for adjustment. Patches were rendered in randomly chosen colors.

Dual channel airborne LiDAR system AX80, developed by Trimble, is a new generation airborne LiDAR designed to provide rapid and efficient point cloud acquisition. One laser emitter is directed in a slightly forward-facing position, with the other facing slightly backward when collecting data. Thus, two strips of the same region are collected at the same time in different views [51]. As with a single channel LiDAR system, small discrepancies still exist between adjacent strips even if careful manual calibration is performed. The present experiment was to validate the effectiveness of the proposed method for strip adjustment of the data acquired by AX80. Experimental data collected in the city of Shi Jiazhuang, Hebei Province, China in 2014 was used in the experiment. The average flying height is about 2300 m, average flying speed is 72 m/s, and average strip width is 1900 m. The average point density is approximately 2.5 points/m^2^ and the average point distance is approximately 0.6 m. Due to the special structure of laser scanner, lateral overlap between strips obtained by two lasers are almost 100%. Main objects in the scene include buildings, trees and roads, as shown in Figure 5a for two adjacent strips. System calibration has been carried out on the original data.

#### 3.2.2. Results of Building Matching and Planar Patch Segmentation

The same processes as the first experiment were applied to extract building footprints from the experimental dataset, as shown in Figure 5b with red and white representing laser points from adjacent strips. Figure 5b shows that the regions covered by the two strips are almost the same due to the dual channel configuration of AX80. Individual buildings segmented were displayed in Figure 5c, where points belonging to the same building were rendered with the same color. More buildings were segmented when compared to the results of experiment one. To reduce redundant calculations and improve algorithmic efficiency, buildings with 100 to 500 points were used in the process of building matching, in which the MHD was set to 10 m. The extracted building pairs were displayed in Figure 5d. The same parameters as experiment one were used for roof plane segmentation and matching, except that only roof planes with 50–200 points were adopted for plane matching. For the two strips displayed in Figure 5a, 79 pairs of corresponding planes were detected from the matched buildings, as shown in Figure 5e.

#### 3.2.3. Final Results

Parameters for the adjustment model were estimated in a similar manner as in experiment one. Profile results, RMSE and $N_{RMSE}$ before and after adjustment were listed in Table 4 for the adjacent strips shown in Figure 5a. It is obvious from Table 4 that discrepancies between adjacent strips were mostly removed after the strip adjustment by the proposed method. The average $D_{mean}$ decreases from 0.258 m to 0.009 m by the adjustment, which slightly outperforms the comparison method. Moreover, the $N_{RMSE}$ is increased by 0.4% in the proposed method, indicating the effectiveness of our method for strip adjustment for the dual channel LiDAR system.

### 3.3. Discussion on the Pixel Size of the Binary Image

**Figure 6:**
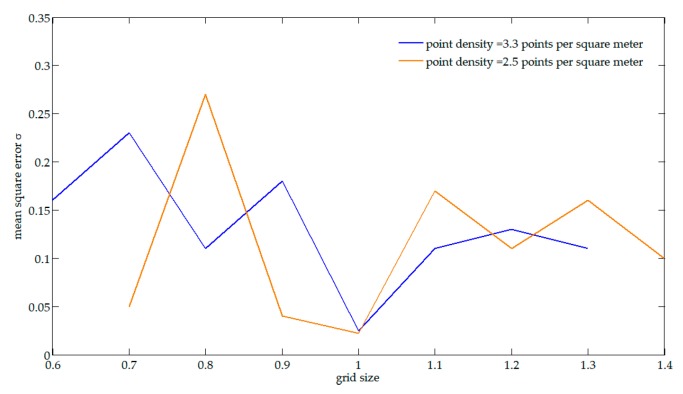
Relationship between the grid size and the mean square errors.

Building segmentation is one of the key steps in the proposed method. It is based on the segmentation of the binary image where binary 1 indicates the current pixel contains building points. The most important factor affecting the generated binary image is the size of the grid cell based on which the point cloud is converted to an image. A too small grid size can break down a building into several separated parts, and the number of the parts in one strip may differ from that in the adjacent strip, which can lead to a failed building matching. A too large grid size, on the other hand, can merge adjacent buildings into one, which also can lead to a failed matching. The optimal size of the grid is determined by multiple related factors, and the density of the point cloud is the principal one. In [52], a more specific definition of density for building recognition was proposed and it was pointed out that an average density of 1 point/m^2^ can detect a building roof sized 2.8 m × 2.8 m. Furthermore, an experiment was conducted to indicate the relationship between the grid size and the mean square errors σ given the average point density of the two datasets, as shown in Figure 6. It can be seen that too small or large grid size both lead to the failure of parameters estimation. A number of corresponding planar patches can be detected, and parameters are correctly calculated if the grid size is set can be set optimally. However, the mean square errors σ do not linearly increase with grid size as expected. As pointed out in [17,21], if the detected planar patches for adjustment have similar orientation and slope, then the accuracy of adjustment may be reduced conversely. In other words, the orientation, slope and distribution of the corresponding, as well as the size of grid jointly influence the errors. From the results of Figure 6, we concluded that the optimal grid size was one meter for our two datasets in the experiments.

### 3.4. Discussion on the Order of Strip Pairs for Adjustment

**Figure 7:**
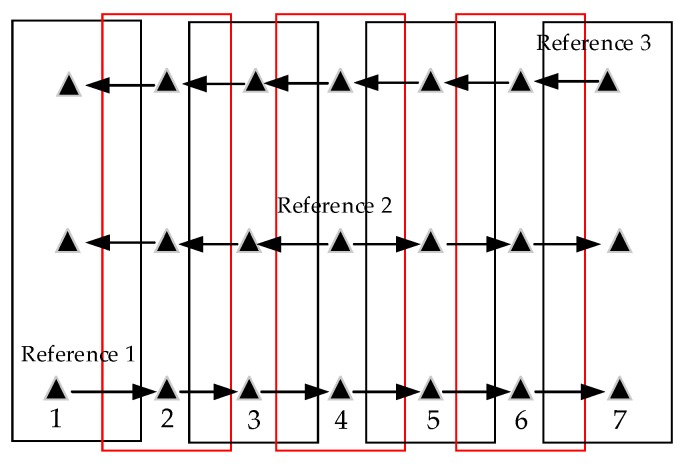
Three types of order of strip pairs for adjustment.

**Figure 8:**
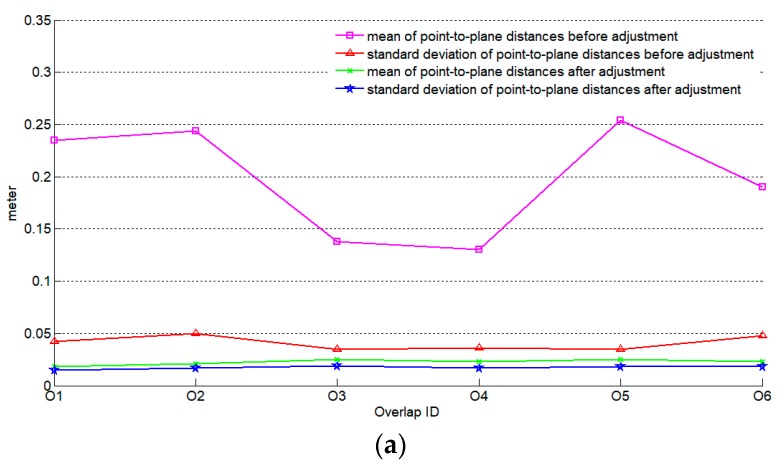

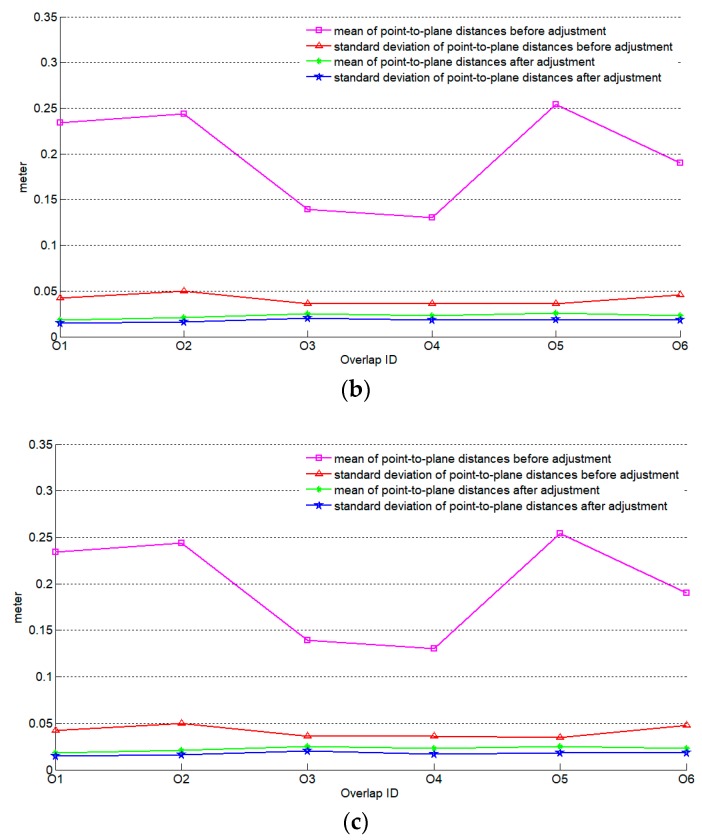
The influence of order of strip pairs on adjustment evaluated by mean and standard deviation of point-to-plane distances: (**a**) Results of type one order; (**b**) Results of type two order; (**c**) Results of type three order.

In terrestrial laser scanning, the accuracy of multi-station registration is influenced by the order of individual stations [53,54]. Likewise, it is meaningful to study whether the order of strip pairs for adjustment affects the final results. Seven strips were chosen from the single channel LiDAR dataset and three types of order were defined: In type one order, the first strip was selected as a reference one. Then, conducted adjustment process strip by strip successively. In type two and type three order, the middle and the last strip were selected as the reference ones, respectively, as shown in Figure 7. For an area with multiple strips, the first strip is defined as the first flight that an airborne LiDAR begins to collect data. Mean and standard deviation of point-to-plane distances were adopted as indicators for evaluation, which were illustrated in Figure 8.

**Figure 9:**
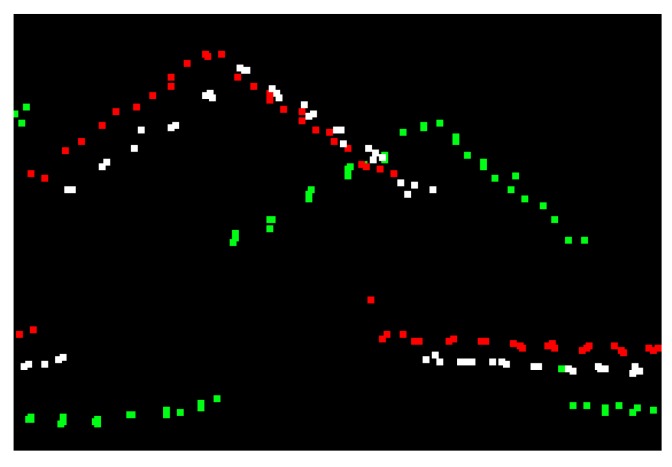
Overlaying a profile of a building roof containing points obtained by adjustment with three types of strip-pair order (red, white and green points were from type one, type two and type three respectively.).

Figure 8 shows that the mean and standard deviation of point-to-plane distances between adjacent strips can effectively be reduced no matter which type of strip-pair order is applied in the adjustment, indicating that it is unlikely that the order of the strip-pairs will influence the adjustment accuracy. Thus, in an ideal case, results of different adjustment orders should match each other perfectly. However, Figure 9 shows that obvious discrepancies exist, which is understandable because reference strips are different among these three scenarios. This is an interesting phenomenon and probably can be exploited for further refinement of the adjustment result, for instance, by minimizing the discrepancies.

## 4. Discussion

Due to the irregular nature of the LiDAR data, one of the most important steps in the process of strip adjustment is the detection of corresponding features between adjacent strips [17,18,22,23,24]. However, large numbers of corresponding planar patches can be detected in an automatic manner in the proposed method, which is of significance for strip adjustment. Building matching and planar patches matching are two core steps in this process. In former step, corresponding buildings in adjacent strip are matched based on MHD. In latter step, MHD and normal vectors similarity are both used to match patches from corresponding buildings. After above steps, corresponding planar patches are automatically detected. From the experimental results of two different types of dataset, numbers of planar patches were detected, and discrepancies between adjacent strips were eliminated. It demonstrates that corresponding planar patches can be detected by introducing MHD in matching process. However, in comparison method [17], compared with our method, corresponding planar patches are selected manually, which is time-consuming.

Despite more planar patches can be detected from corresponding buildings by the proposed method compared with the method in [17], some corresponding planes, especially those with similar orientations and slopes in a local region, are unnecessary for strip adjustment [17,19,21,47]. As pointed out in [21,47], if the detected corresponding planar patches for adjustment estimation have similar orientation and slope, then the accuracy of the adjustment may be reduced, whilst the patches in different orientations and slopes can reduce the correlation among the estimated parameters. Thus, planes with different orientations and slopes are preferred in adjustment calculation [17,19]. Moreover, [21] showed even distribution of slopes in different directions reduced the correlation among the estimated parameters, either. Therefore, we believe that our method will perform better in terms of accuracy and efficiency if evenly distributed corresponding planar patches with different orientations and slopes and even distribution are selected for the adjustment calculation. This is a problem which deserves further studies.

In addition, coarse-to-fine roof segmentation method is completely based on the properties of a plane, which is widely adopted in roof segmentation [28,43,55]. Though it can detect enough patches for adjustment, several predefined parameters are required, which limit the automation level of the proposed method. A more automatic method for planar patch detection to aid in strip adjustment remains a challenge. Moreover, there are other distance available, such as Euclidean distance, Manhattan distance, Chebyshev distance, etc. [56,57], but only Euclidean distance was used in the calculation of Hausdorff Distance (HD). How to introduce other distances to the HD calculation and its potential influence to building and roof plane matching are worth further research.

## Figures and Tables

**Table 1:** Comparison of the proposed method and the one from literature [17].

Aspects	The Proposed Method	The Comparison Method
R(φ,ω,κ)	$\left[\begin{array}{ccc} 1.000629 & 0.000034 & 0.012337\\ 0.000366 & 1.001069 & -0.029612\\ -0.000542 & -0.000011 & 0.997252 \end{array}\right]$	$\left[\begin{array}{ccc} 0.999984 & 0.000697 & -0.044519\\ -0.002924 & 0.999815 & -0.074462\\ -0.000441 & 0.000125 & 0.993573 \end{array}\right]$
T	$\left[\begin{array}{c} -0.211\\ -0.358\;\\ -0.248 \end{array}\right]$	$\left[\begin{array}{c} -0.014\\ 0.198\\ -0.155 \end{array}\right]$
σ (m)	0.023	0.025
Number of corresponding planes	114	8
Manner of selecting corresponding planes	Automatic	Manual

**Table 2:** Visual and quantitative comparison of adjustment of two adjacent strips.

Before Adjustment Profiles $D_{mean}$		Proposed Method Profiles $D_{mean}$		Comparison Method Profile $D_{mean}$		TerraMatch Profile $D_{mean}$	
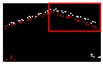	0.353	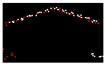	0.011	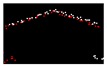	0.023	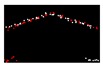	0.013
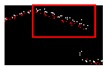	0.358	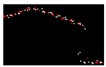	0.011	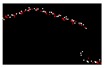	0.013	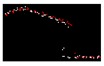	0.023
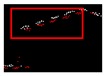	0.423	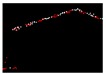	0.007	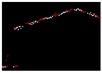	0.010	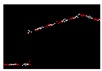	0.006
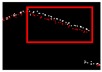	0.457	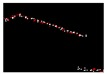	0.008	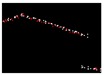	0.007	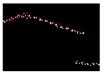	0.011
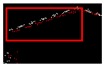	0.375	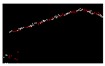	0.007	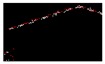	0.010	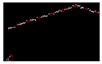	0.005
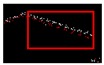	0.346	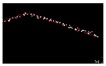	0.006	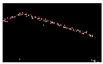	0.006	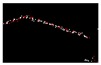	0.005
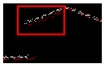	0.510	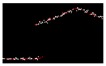	0.012	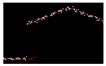	0.013	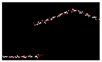	0.015
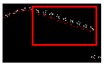	0.478	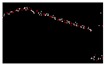	0.012	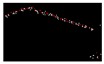	0.011	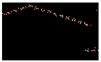	0.014
RMSE	0.417	- -	0.010	- -	0.013	- -	0.013
$N_{RMSE}$	- -	- -	97.6%	- -	96.8%	- -	96.8%

**Table 3:** Results of the strip adjustment to the six pairs of strips.

Serial No. of Adjacent Strips	Number of Corresponding Buildings	Number of Corresponding Roof Planes	σ(m)
1 & 2	70	114	0.023
2 & 3	37	54	0.028
3 & 4	102	118	0.035
4 & 5	128	171	0.030
5 & 6	121	152	0.032
6 & 7	36	45	0.033

**Table 4:** Visual and quantitative comparison of adjustment of two adjacent strips.

Before Adjustment Profiles $D_{mean}$		Adjustment by the Proposed Method Profiles $D_{mean}$		Adjustment by the Comparison Method Profiles $D_{mean}$	
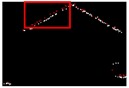	0.212	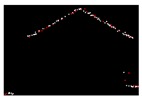	0.011	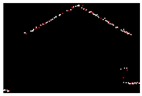	0.012
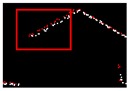	0.322	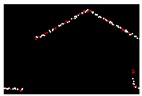	0.007	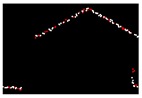	0.007
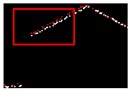	0.206	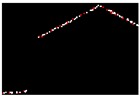	0.011	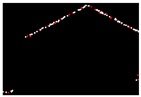	0.013
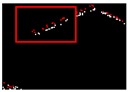	0.253	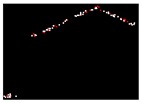	0.009	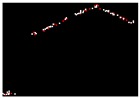	0.011
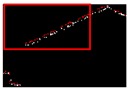	0.197	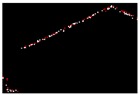	0.013	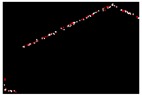	0.013
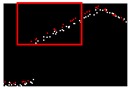	0.222	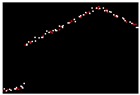	0.008	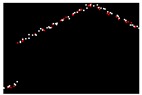	0.009
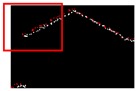	0.230	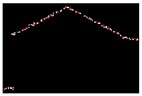	0.012	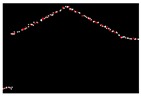	0.014
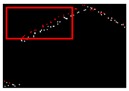	0.422	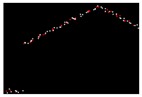	0.004	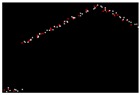	0.007
RMSE	0.268	- -	0.010	- -	0.011
$N_{RMSE}$	- -	- -	96.3%	- -	95.9%
